# Acceleration profiles and processing methods for parabolic flight

**DOI:** 10.1038/s41526-018-0050-3

**Published:** 2018-08-07

**Authors:** Christopher E. Carr, Noelle C. Bryan, Kendall N. Saboda, Srinivasa A. Bhattaru, Gary Ruvkun, Maria T. Zuber

**Affiliations:** 1 Department of Earth, Atmospheric and Planetary Sciences, Massachusetts Institute of Technology, Cambridge, MA USA; 2 Department of Molecular Biology, Massachusetts General Hospital, Boston, MA USA; 3 Department of Aeronautics and Astronautics, Massachusetts Institute of Technology, Cambridge, MA USA

## Abstract

Parabolic flights provide cost-effective, time-limited access to “weightless” or reduced gravity conditions, facilitating research and validation activities that complement infrequent and costly access to space. Although parabolic flights have been conducted for decades, reference acceleration profiles and processing methods are not widely available. Here we present a solution for collecting, analyzing, and classifying the altered gravity environments experienced during parabolic flights, which we validated during a Boeing 727-200F flight with 20 parabolas. All data and analysis code are freely available. Our solution can be integrated with diverse experimental designs, does not depend upon accelerometer orientation, and allows unsupervised classification of all phases of flight, providing a consistent and open-source approach to quantifying gravito-inertial accelerations (GIA), or *g* levels. As academic, governmental, and commercial use of space advances, data availability and validated processing methods will enable better planning, execution, and analysis of parabolic flight experiments, and thus facilitate future space activities.

## Introduction

**Fig. 1:**
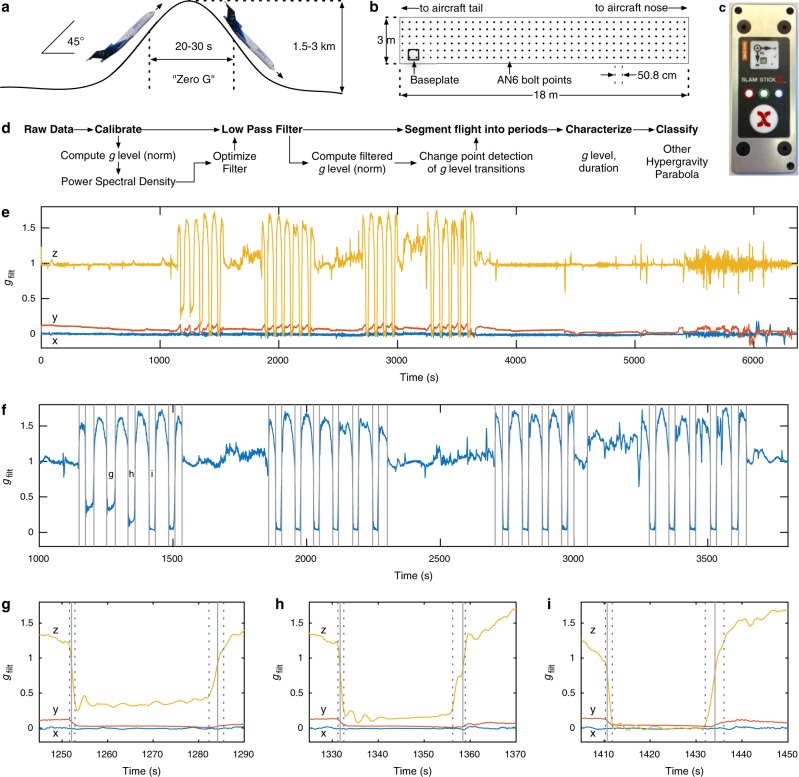
Parabolic flight acceleration data and analysis methodology. **a** Typical flight path during a single parabola. **b** Research section of aircraft with baseplate location during flight. **c** Accelerometer orientation on baseplate. **d** Overview of analysis method. **e** Measured accelerations after low-pass filtering (*g*_filt_). **f** Change points (vertical lines) for mean *g* levels as measured by *g*_filt_. **g**–**i** Second-level linear change points (vertical dotted lines) define transition regions for a Mars, a lunar, and a 0 *g* parabola, respectively. Individual parabolas corresponding to **g**–**i** are labeled in **f**. For accelerations during each of the 20 parabolas, see Supplementary Figs. 6–7

Parabolic flights are cost-effective, ground-based analogs that achieve variable *g* (*Earth-relative GIA*) level environments that recreate conditions experienced during space flight.^1,2^ Specialized aircraft can maintain approximately 20–30 s of a 0 *g*, freefall environment before an increased GIA recovery phase (Fig. 1a). Modified trajectories can achieve reduced *g* levels experienced on the lunar surface or on Mars (0.17 and 0.38 *g*, respectively). Parabolic flights serve as valuable proving grounds for experimental efforts to maximize the research potential of the International Space Station^3,4^ and to accommodate increasing interest in commercial space flight.^5,6^

Here we address the limited availability of open access acceleration datasets containing parabolic flight profiles and enable unsupervised and precise characterization of timing and *g* levels for all flight phases. We demonstrate this approach using a small (65 g) battery powered commercially available accelerometer and vibration measurement system. Together, these tools and products reflect a comprehensive solution for experiment planning, execution, and analysis of *g* level and vibrations during parabolic flight.

## Results

Flight operations were conducted on November 17, 2017 onboard a Boeing 727-200F aircraft (G-Force One®, Zero Gravity Corporation). Four sets of parabolas were performed with 5, 6, 4, and 5 parabolas, respectively. The first set targeted, in order, Mars *g*, Mars *g*, Lunar *g*, 0 *g*, and 0 *g*. All other parabolas targeted 0 *g*. Data were collected for 1.77 h during all phases of flight from a Slam Stick X™ (Mide Technology Corp.) mounted in the rear of the research section (Fig. 1b-c). Direct Current (DC) acceleration was recorded at 411 Hz. Additional data, calibration, and calibration error ( < 2%) assessment are described in Methods.

In any given experiment, one accelerometer orientation may be more appropriate than another. Thus, our phase of flight identification is based on a metric that is independent of accelerometer orientation: the Euclidean norm of the accelerometer (x,y,z) axes, which we refer to as *g* level or *g* (Methods).

To facilitate our analysis (Fig. 1d, Methods, Supplementary Figs. 1-5), acceleration data were filtered (Fig. 1e) using a zero-delay, low-pass filter prior to parabola identification using change point detection.^7,8^ Conceptually, this process finds the point for which a statistical property (e.g., mean), has minimum total residual error summed across two groups, e.g., before and after the change point. Here residual error is the difference between an observed value and the statistical property for the group.

**Fig. 2:**
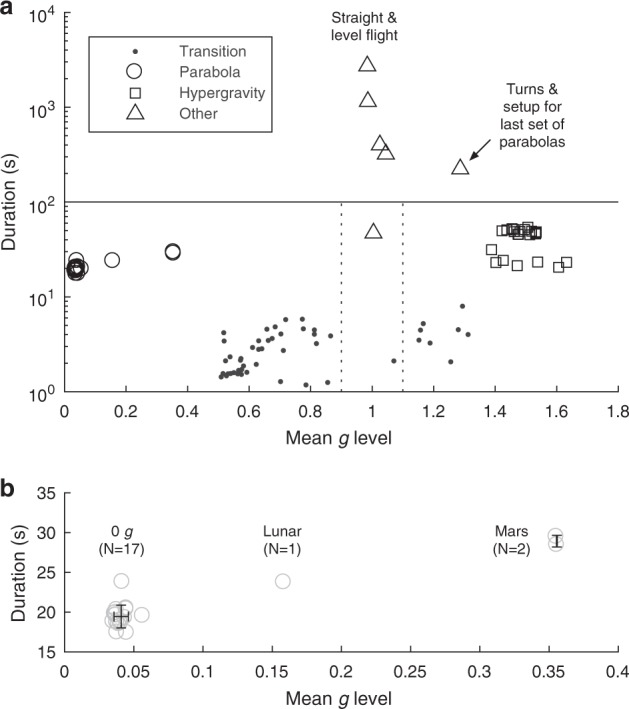
Phases of flight classification and characterization. **a** After identifying transitions, non-transition events were classified as duration < 100 s (horizontal line) and by mean *g* level (vertical dotted lines) into “parabola,” “hypergravity,” and “other.” The subset of “hypergravity” periods with lower duration were identified as those at the start or end of a set of parabolas. All phases of flight were unambiguously classified. **b** Parabola characteristics (see also: Supplementary Information). Error bars: mean ± SD

Change point detection was first applied to the filtered *g* level, *g*_filt_, to identify differences in mean *g* levels in an unsupervised manner (Fig. 1f). To break down the flight into regions of stable *g* levels, data within 10 s of each change point was subjected to secondary change point detection using a linear slope metric, which segmented the flight into regions of rapid “transition” (indicated by dotted lines, Fig. 1g-i) and more stable regimes. Non-transition periods were subsequently classified into “parabola,” “hypergravity,” and “other” based on their duration and *g* level (Fig. 2a-b; Methods). These “hypergravity” periods result from entry into and exit from the “parabola” periods.

## Discussion

Parabolic flights provide the opportunity to perform simulated space research in a cost-effective manner. Recently, Lambot and Ord (2016) evaluated data from over 400 parabolic flights and assessed the quality of reduced *g* datasets.^9^ Although considerable effort was dedicated to identifying the highest quality, low *g* time periods (with variations less than ± 0.01 *g*) from these flights, neither the acquisition hardware, the raw data, nor the code implemented for analysis, are currently available to the public. Indeed, when reviewing the literature, we found no such easily accessible parabolic flight acceleration data nor published analysis methodology.

Here, we provide the following: (1) a commercially available hardware solution for data acquisition; (2) raw and calibrated data for all phases of flight; (3) data analysis methodology that is independent of accelerometer orientation, and (4) characterization of *g* levels and durations achieved for 20 parabolas. In addition, the code implementing our methodology to categorize all phases of flight and characterize *g* levels and durations of parabolas is publicly available in order to facilitate future parabolic flight research. In addition, our methods could be adapted to analysis of data from suborbital flights, drop towers, or studies involving launch and landing accelerations.

Our hardware solution, the Slam Stick X™, offers a compact, flexible, low-power, high resolution solution for acceleration and vibration monitoring with favorable comparison to alternatives (Methods). The small size facilitates integration into experiments and measurement of the local GIA environment, which is not constant across the aircraft. In addition, mounting with double-sided tape is simple, robust, and does not impact the frequency response (Methods).

Lambot and Ord^9^ identified a “sweet zone” in the middle of the parabola with low acceleration deviation. Our data are consistent with this conclusion (Supplementary Fig. 8), although Lambot and Ord^9^ used, after unspecified low-pass filtering, a much more stringent tolerance (± 0.01 *g*) that was not met by our filtered data. We encourage future parabolic flight experimenters to release raw data as well as to provide data processing details (ideally including in executable form) to facilitate validation and improvement of processing algorithms, as well as refine expected *g* levels and support planning and analysis that can be tailored to specific experimenter needs.

Due to the limited availability and high cost of actual space environments, it is imperative that we continue to utilize parabolic flights as a means to simulate space – and to understand the accuracy and limitations of this modality. By making our data and methods available we hope to enable others to better plan, execute, and analyze parabolic flight experiments, and thus to help facilitate future space activities.

## Methods

### Device Selection

The Slam Stick X™ (Mide Technology Corp., www.mide.com) was selected based on its size (76 mm × 30 mm × 15 mm), low mass (65 g), integrated battery, manual and USB interfaces, and combination DC (Analog Devices ADXL345) and piezoelectric (TE 832M1) accelerometers to enable accuracy at both low (e.g., down to 0 Hz) and high frequencies (up to 20 kHz sample frequency). The aluminum body was selected to provide improved high frequency response. Additional integrated sensors included temperature and pressure (NXP MPL3115) and control pad temperature and pressure (TE MS8607).

Alternative data acquisition systems include many commercial off the shelf (COTS) accelerometers, as well as the NASA Suborbital Flight Environment Monitor (SFEM)^9^. There may be potential benefits of using the SFEM, although the Slam Stick X™ offers comparable or longer recording time, DC and piezoelectric accelerometers (enabling both *g* level and high frequency vibration measurements), higher sampling frequencies, a wider operating temperature range (− 40 °C to + 80˚C) and much lower ( > 10 × ) mass and volume. Another COTS option is the Lansmont 3X90  , although the Slam Stick X™ specifications provide benefits in several areas (size, mass, sampling rates, and temperature range).

One consideration for parabolic flight experiments is that the GIA environment is not constant across the aircraft. In some cases, it may be adequate to have a single reference flight profile to be used by multiple experiment teams. However, some applications may be better served through measurement of the local GIA environment of a given experimental apparatus. Here, the small size of our solution facilitates direct incorporation into a payload, as well as placement in the desired location or orientation.

When selecting a data acquisition solution, it is also important to consider how the mounting of the accelerometer itself may impact the frequency response; in our case, use of double-sided sticky tape represents both an extremely practical and low bias option, enabled by the low device mass. Because no additional materials separate the accelerometer from the aircraft, there is no need to correct for the frequency response of the mounting interface.

### Device mounting and data acquisition

Zero Gravity Corporation (ZGC) utilizes a standard system of mounting hardware to the aircraft structure consisting of a baseplate (61 cm × 61 cm × 1.27 cm aluminum plate, e.g., McMaster Carr 86825K25) bolted to the aircraft structure using four clearance holes at the corners of a square with 50.8 cm (20 in) sides, centered on the baseplate. Washers (McMaster Carr 92503A230) were used for mounting in combination with AN-6 steel bolts (3/8 inch) provided by ZGC. The Slam Stick X™ was mounted to a standard baseplate with double-sided sticky tape (3 M 950), which is the preferred mounting method due to its vibration frequency response (near unity) and robustness: this method has previously been validated during vibration testing at over 75 g at 1 kHz^1^. The Slam Stick X™ was configured using Slam Stick Lab 1.8. Acquisition was initiated and terminated manually using the control pad on the device. Sampling rates were 5 kHz (piezoelectric vibration sensor), 411 Hz (DC acceleration), and 1 Hz (pressure and temperature).

### Data calibration

The raw IDE file generated by the Slam Stick X™ was converted to calibrated MAT (MATLAB, The Mathworks, Natick, MA) files using the ide2csv.exe command line utility (Mide Technology Corp.) using the factory calibration. Note that data calibration and export functions can also be performed directly using Slam Stick Lab. Here we focus on data from the DC accelerometer. We note that temperature varied less than 1 °C and pressure showed a typical regulated profile and was highly stable during parabolas (Supplementary Fig. 5).

### Orientation-independent approach

In any given experiment, one accelerometer orientation may be more appropriate than another. Thus, we based our phase of flight identification method (below) on a measure that is independent of the accelerometer orientation: the Euclidean norm of the x, y, and *z* axes, which we hereafter refer to as the *g* level or *g*. As this variable is a positive scalar, it does not capture directional fluctuations in the gravity vector. Thus, for characterization of phases of flight, vector-based statistics should be used. For example, we estimated the mean g level during a 0 *g* parabola by averaging the *x*, *y*, and *z* components, and then computing the norm.

### Calibration verification

The expected value of the *g* level is unity on Earth under non-accelerated conditions; as a verification of our accelerometer calibration, we found the norm under lab bench conditions (14.2 s recording) to be 0.9840 (rms) and 0.9840 ± 0.0055 (mean ± SD), consistent with < 2% error. This is a lower bound when vibration or specific force other than that caused by gravity is present, consistent with the rms value (1.07) observed during flight. Specific force was concentrated in the *z* axis as measured by root mean square (rms) values (0.0466, 0.0775, 1.0662 for *x*, *y*, and *z* axes, respectively), consistent with the accelerometer orientation (Fig. 1c). Calibration accuracy was also assessed after filtering (see below).

### Phase of flight characterization

For parabola identification, we first filtered the raw data using a zero-phase 12th order Butterworth filter using the *designfilt()* function using a half power frequency (HPF) as described below. Next, we utilized change point detection^7,8^ as implemented by the MATLAB *FindChangePts()* function.

Change point detection was first applied to the filtered *g* level *g*_filt_ to identify differences in mean *g* levels. A known number of change points was specified based on the parabola number within each set, e.g., two times the number of parabolas, plus two additional transitions (first pre-parabola pull up; last post-parabola pull up) for each set of parabolas. In our case sets of 5, 6, 4, and 5 (20 total) parabolas become 12, 14, 10, and 12 change points. This total number of change points (48) was specified and *FindChangePts()* identified all *g* level change points in an unsupervised manner (Fig. 1f).

We desire to break down the flight into regions of stable *g* levels. Thus, for each change point, we used a secondary change point detection to identify differences in the slope of the *g* level vs. time curves. Data within 10 s of each change point was subjected to this secondary change point detection using a linear slope metric. This step successfully segmented the flight into regions of rapid “transition” (indicated by dotted lines, Fig. 1g-i) and more stable regimes. This resulted in 97 flight periods (2 × the number of change points + 1).

Classification of non-“transition” flight periods into “parabola,” “hypergravity,” and “other” (which includes straight and level flight as well as standard rate turns) was then performed, first by categorizing any periods with duration > 100 s as “other”, then by segmenting data according to *g* level (“parabola” ≤ 0.9 *g*, 0.9 < “other” ≤ 1.1 *g*, “hypergravity > 1.1 *g*). Despite its simplicity, this classifier achieved good separation between classes (Fig. 2a).

Parabola durations (mean ± s.d.) were 19.5 ± 1.4 s (0 *g*, *N* = 17, range 17 to 24 s), 23.7 s (Lunar *g*; *N* = 1), and 28.9 ± 0.7 s (Mars *g*; *N* = 2). The *g* levels achieved were 0.041 ± 0.005 *g* (0 *g*) and 0.159 *g* (lunar *g*). Both Mars parabolas achieved 0.356 *g*, indicating high consistency between parabolas targeting similar *g* levels. Higher *g* levels were significantly associated with longer-duration parabolas (Supplementary Fig. 4a), although not when lunar and Mars data were excluded (Supplemental Fig. 4b).

Some limitations are inherent in our study, which focused solely on one flight and 20 parabolas. If analyzing multiple flights, with parabolas performed under more varied conditions, it is possible a slightly more complex classification strategy might be required; however, based on the wide separation between “parabola,” “hypergravity,” and “other” classes, this is not expected to present a significant challenge to standard unsupervised classification approaches (e.g., *k*-means).

### Filter optimization

To optimize the filter, we selected a HPF based on the *g* level power spectral density (PSD, Supplementary Fig. 2a). The PSD was computed via the MATLAB *pwelch()* function with default parameters. To select the HPF, we examined the cumulative sum of the PSD (Supplementary Fig. 2b), which revealed a sharp increase in power above 0.01 Hz. We chose this value (HPF = 0.01 Hz) to maximize the low frequency content of the filtered data while rejecting as much spectral power from higher frequencies as possible. As an example, filtering at HPF = 0.01 Hz preserves parabola dynamics, whereas filtering at HPF = 0.001 Hz does not (Supplementary Fig. 2c). Our selected value provides the smoothest data for identifying parabolas, while still accurately representing *g* level transitions. A manual procedure identified similar values, e.g., adjusting the HPF toward DC until the rapid transitions between *g* levels showed systematic bias, then setting the HPF to 10 × this value, also gave HPF = 0.01 Hz.

Filtering reduced the root mean square specific force in the lateral (x) direction but little in other directions (0.0161, 0.0681, 1.0623 for *x*, *y*, *z*, respectively), consistent with low frequency aircraft accelerations mainly due to pitch maneuvers. The *g* level was near unity during periods of relative calm (Supplemental Fig. 3a-b), including the first 1000 s of data collected during largely straight and level flight (rms 0.9919 and 0.9856, raw and filtered, respectively). This unfiltered estimate is 0.8% higher than under lab bench conditions, and both are consistent with accurate sensor calibration at DC to lower than 2% error, based on the factory calibration.

### Regression analysis

Regression of parabola *g* level on duration was performed using the MATLAB *fitlm()* function. Confidence intervals were determined using the MATLAB *coefCI()* function.

### Code availability

The MATLAB scripts implementing our analysis are available at: https://github.com/CarrCE/zerog.

### Data availability

Raw and calibrated data are available via the Open Science Framework at: https://osf.io/nk2w4/.

## Electronic supplementary material

Supplementary Information: Acceleration Profiles and Processing Methods for Parabolic Flight

## Footnotes

1 Mide Technology Corp., Slam Stick User Manual, Version 2.0

## Electronic supplementary material

**Supplementary information** accompanies the paper on the *npj Microgravity* website (10.1038/s41526-018-0050-3).

## References

[1] Karmali F, Shelhamer M (2008). The dynamics of parabolic flight: flight characteristics and passenger percepts. Acta Astronaut..

[2] Shelhamer M (2016). Parabolic flight as a spaceflight analog. J. Appl. Physiol..

[3] Rai A (2016). Expanded benefits for humanity from the International Space Station. Acta Astronaut..

[4] Shelhamer, M. Why send humans into space? Science and non-science motivations for human space flight. *Space Policy*, 10.1016/j.spacepol.2017.10.001 (2017).

[5] Smith L (2017). Commercial space flight. Congr. Dig..

[6] Musk E (2017). Making humans a multi-planetary species. New Space.

[7] Lavielle M (2005). Using penalized contrasts for the change-point problem. Signal Process..

[8] Killick R, Fearnhead P, Eckley IA (2012). Optimal detection of change points with a linear computational cost. J. Am. Stat. Assoc..

[9] Lambot, T. & Ord, S. F. Analysis of the quality of parabolic flight. *Next-Generation Suborbital Researchers Conference*, https://ntrs.nasa.gov/search.jsp?R=20160007932 (2016).

