# Supplementary material for: Acceleration profiles and processing methods for parabolic flight
Source: NPJ Microgravity. 2018 Aug 7;4:14. doi: 10.1038/s41526-018-0050-3 (PMC6081456; doi:10.1038/s41526-018-0050-3)
Supplement: Supplementary file 1 — Supplementary Information: Acceleration Profiles and Processing Methods for Parabolic Flight [file 41526_2018_50_MOESM1_ESM.pdf]

Supplementary Information: Acceleration Profiles and Processing Methods for Parabolic Flight

Christopher E. Carr[1,2,*], Noelle C. Bryan[1], Kendall N. Saboda[1], Srinivasa A. Bhattaru[3], Gary Ruvkun[2], Maria T. Zuber[1]

[1]Massachusetts Institute of Technology, Department of Earth, Atmospheric and Planetary Sciences, Cambridge, MA, USA. [2]Massachusetts General Hospital, Department of Molecular Biology, Boston, MA, USA. [3]Massachusetts Institute of Technology, Department of Aeronautics and Astronautics, Cambridge, MA, USA

*Correspondence: 77 Massachusetts Ave Room 54-418, Cambridge MA 02138, USA. chrisc@mit.edu, +1-617-253-0786.

Here we provide supplementary figures (8).

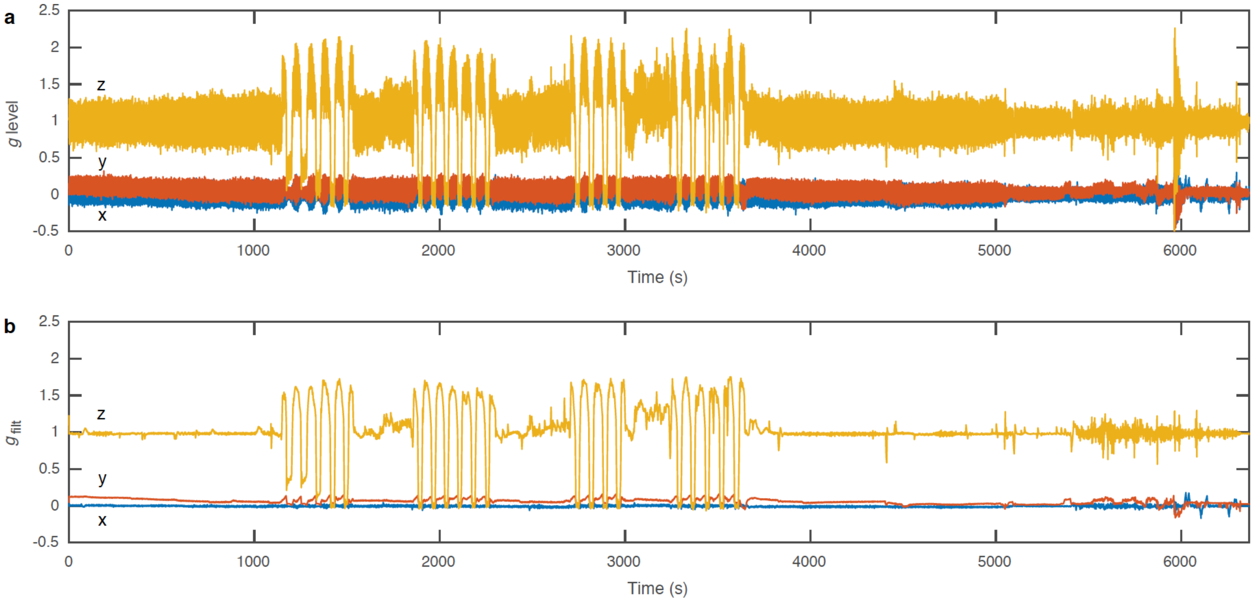

Supplementary Fig. 1. Acceleration profile of a parabolic flight. a Calibrated DC accelerometer data. **b** After low-pass filtering.

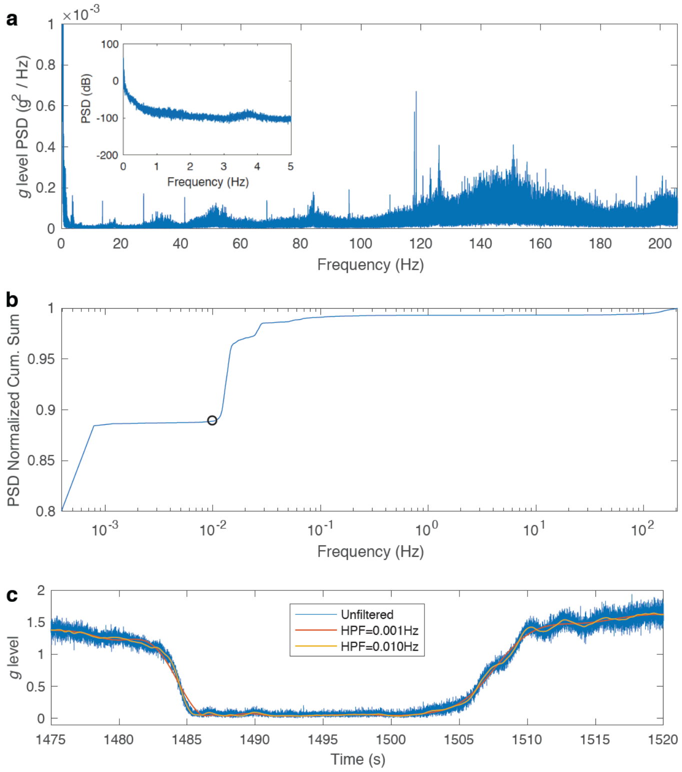

Supplementary Fig. 2. Power Spectral Density (PSD) of *g* level enables rational selection of filter Half Power Frequency. a PSD estimated using Welch's method. Near DC frequencies hold much of the spectral power (inset). **b** Cumulative sum of PSD normalized to unity with selected filter HPF of 0.01 Hz (circle). **c** The *g* level filtered with HPF (0.01 Hz, orange) better matches the unfiltered *g* level (blue) than does a 10x lower frequency HPF (0.001 Hz, red).

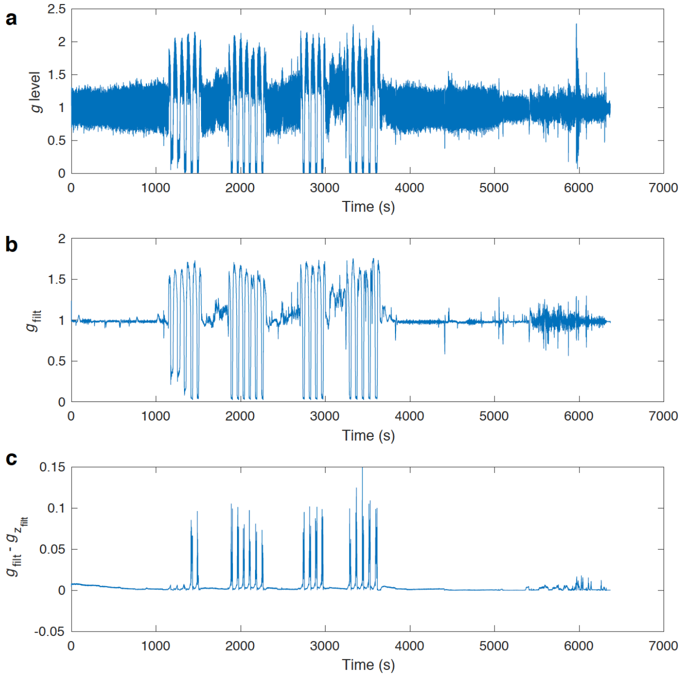

Supplementary Fig. 3. Acceleration profile as measured by *g* level, the norm of the gravity vector. a Unfiltered *g* level. **b** Low-pass filtered *g* level. **c** Difference between *g* level and magnitude of z-axis acceleration g_z. This difference is appreciable during 0 *g* parabolas but not Mars or lunar *g* parabolas, suggesting significant contributions to *g* from x and y axes during zero *g* parabolas. Accelerometer axes are as described in **Fig. 1c**.

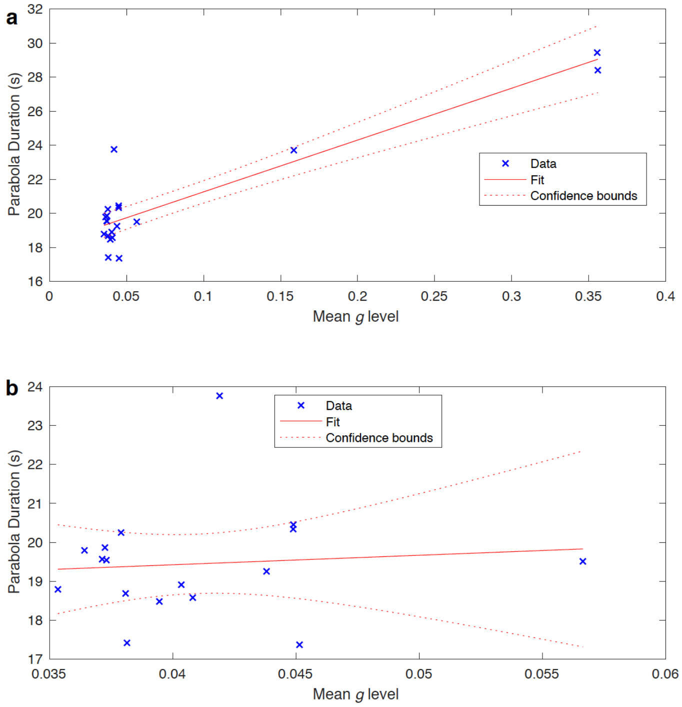

Supplementary Fig. 4. Parabola duration as a function of *g* level. a Regression of *g* level on parabola duration (*N*=20) yielded a highly significant relationship (F=91.3, dof=18, $p<10^{-7}$, one-sided by definition). **b** However, a regression without the limited Lunar *g* (*N*=1) and Mars *g* (*N*=2) data was insignificant (F=0.117, dof=15, *p*=0.737). Thus, care should be taken not to over-interpret the measured relationship.

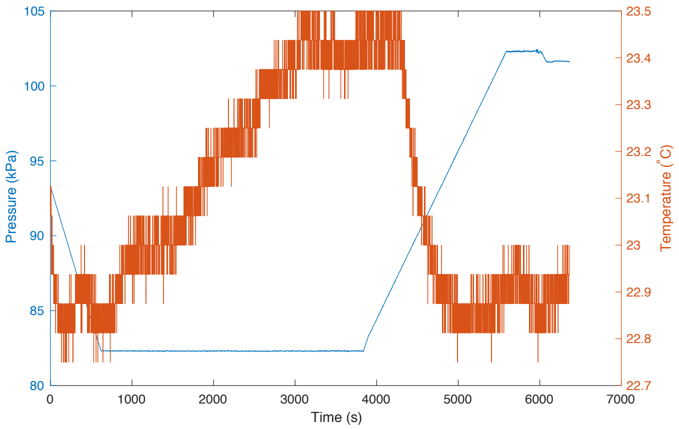

Supplementary Fig. 5. Pressure and temperature during flight. Pressure profile (blue line) reflects pressure altitude of ~1720 m established during flight until completion of parabolas. Measured temperature varied within a tight range around 23˚C.

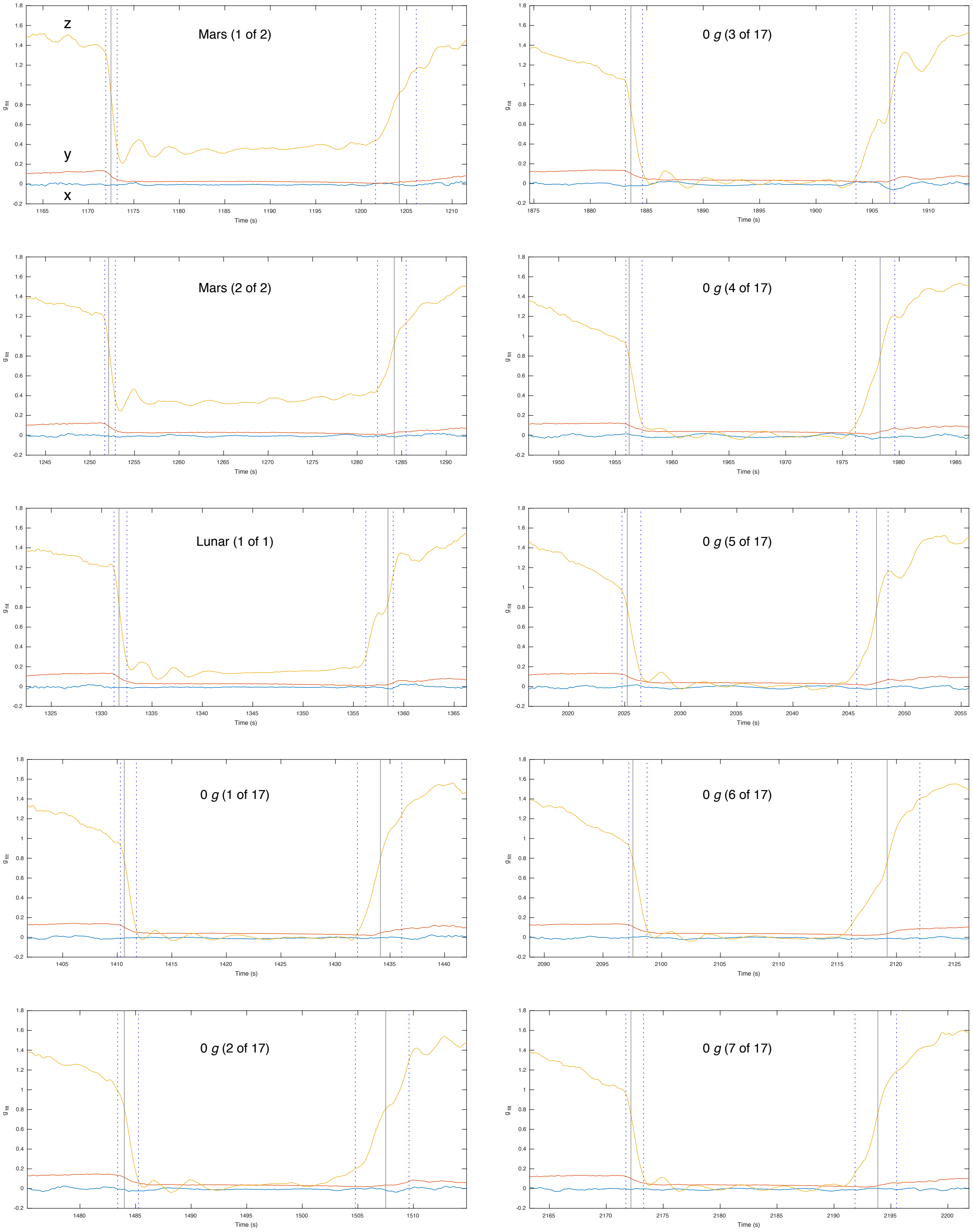

Supplementary Fig. 6. Low-pass filtered accelerations during the first ten parabolas.

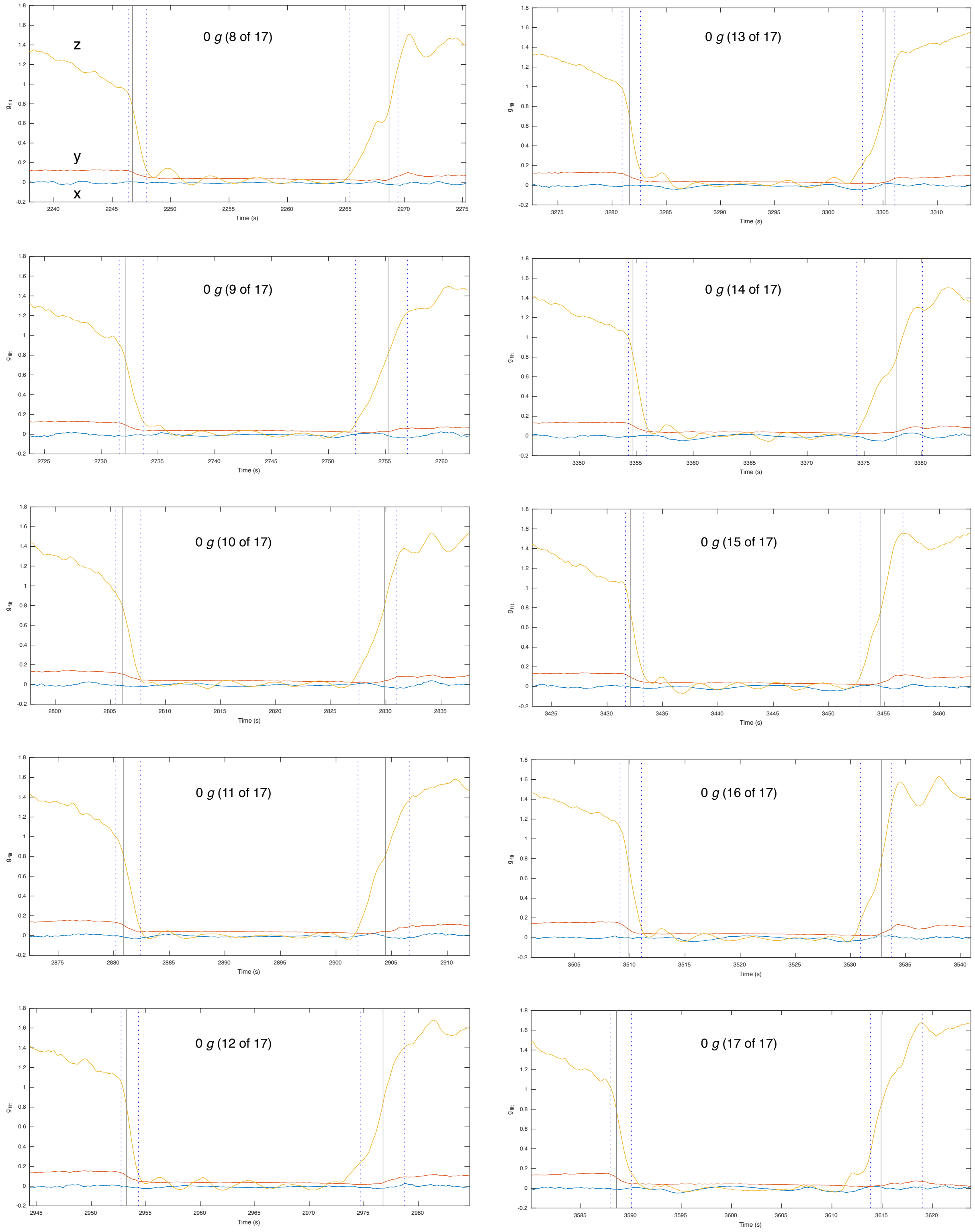

Supplementary Fig. 7. Low-pass filtered accelerations during the second ten parabolas.

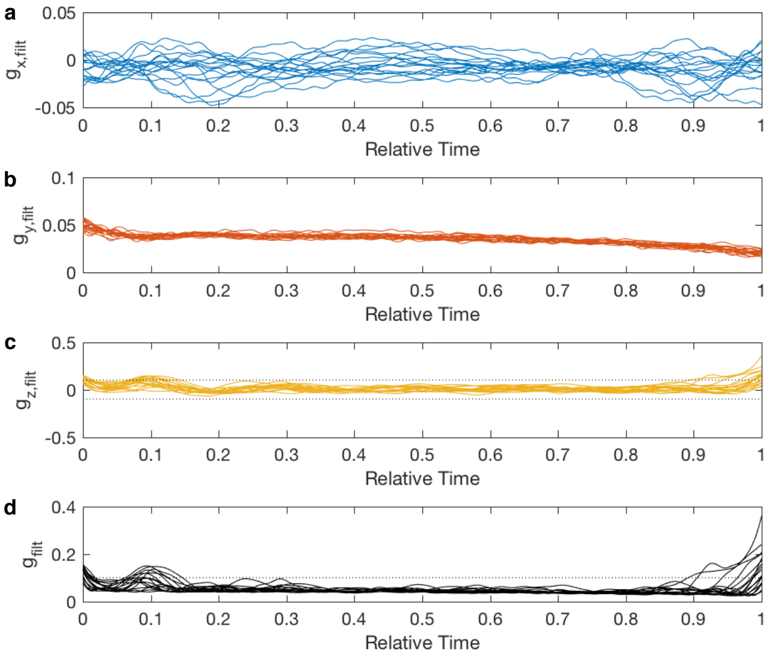

Supplementary Fig. 8. Low-pass filtered accelerations across 0 *g* parabolas. a x-axis. **b** y-axis. **c** z-axis. **d** *g* level (norm).
